# Ionic Polyureas—A Novel Subclass of Poly(Ionic Liquid)s for CO_2_ Capture

**DOI:** 10.3390/membranes10090240

**Published:** 2020-09-18

**Authors:** Sofia M. Morozova, Elena I. Lozinskaya, Haritz Sardon, Fabian Suárez-García, Petr S. Vlasov, Régis Vaudemont, Yakov S. Vygodskii, Alexander S. Shaplov

**Affiliations:** 1 A.N. Nesmeyanov Institute of Organoelement Compounds Russian Academy of Sciences (INEOS RAS), Vavilov Str. 28, 119991 Moscow, Russia; sofiionova@yandex.ru (S.M.M.); helloz@ineos.ac.ru (E.I.L.); yasvyg@ineos.ac.ru (Y.S.V.); 2 Laboratory of Solution Chemistry of Advanced Materials and Technologies, ITMO University, Lomonosova str. 9, 191002 St. Petersburg, Russia; 3 POLYMAT, University of the Basque Country UPV/EHU, Joxe Mari Korta Center, Avda. Tolosa 72, 20018 Donostia-San Sebastian, Spain; 4 Instituto de Ciencia y Tecnología del Carbono, INCAR-CSIC, Francisco Pintado Fe 26, 33011 Oviedo, Spain; fabian@incar.csic.es; 5 Department of Macromolecular Chemistry, Saint-Petersburg State University, Universitetsky pr. 26, 198504 Saint-Petersburg, Russia; petr_vlasov@mail.ru; 6 Luxembourg Institute of Science and Technology (LIST), 5 avenue des Hauts-Fourneaux, L-4362 Esch-sur-Alzette, Luxembourg; regis.vaudemont@list.lu

**Keywords:** poly(ionic liquid)s, ionic polyureas, ionic polyurethanes, CO_2_ capture, solid adsorbent

## Abstract

The growing concern for climate change and global warming has given rise to investigations in various research fields, including one particular area dedicated to the creation of solid sorbents for efficient CO_2_ capture. In this work, a new family of poly(ionic liquid)s (PILs) comprising cationic polyureas (PURs) with tetrafluoroborate (BF_4_) anions has been synthesized. Condensation of various diisocyanates with novel ionic diamines and subsequent ion metathesis reaction resulted in high molar mass ionic PURs (M_w_ = 12 ÷ 173 × 10^3^ g/mol) with high thermal stability (up to 260 °C), glass transition temperatures in the range of 153–286 °C and remarkable CO_2_ capture (10.5–24.8 mg/g at 0 °C and 1 bar). The CO_2_ sorption was found to be dependent on the nature of the cation and structure of the diisocyanate. The highest sorption was demonstrated by tetrafluoroborate PUR based on 4,4′-methylene-bis(cyclohexyl isocyanate) diisocyanate and aromatic diamine bearing quinuclidinium cation (24.8 mg/g at 0 °C and 1 bar). It is hoped that the present study will inspire novel design strategies for improving the sorption properties of PILs and the creation of novel effective CO_2_ sorbents.

## 1. Introduction

The anthropogenic emission of CO_2_, particularly from fossil fuel combustion, is one of the main sources of greenhouse gas emission and global warming [1,2]. The search for effective methods to address the effects of climate change due to increased CO_2_ emissions relates to important challenges facing the global chemical community [2,3].

Purification of flue gas streams can add important value to the fight against CO_2_ emissions. Recently, among other promising approaches, the application of polymeric ionic liquids or poly(ionic liquid)s (PILs) as potential solid sorbent materials for CO_2_ capture and separation gained significant attention [4]. Such attention was deserved due to the fact that PILs, being a subclass of polyelectrolytes, combine the advantages of polymers (processability, film-forming properties, solid state, light weight, etc.) and ionic liquids (high thermal and electrochemical stabilities, enhanced ionic conductivity, high gas absorption, affinity to CO_2,_ etc.) [4,5,6,7,8,9,10,11]. The broad variety of PILs that can be prepared using countless combinations of cations (ammonium, pyridinium, imidazolium, phosphonium, 1,2,3- and 1,2,4-triazolium…) and anions (halide, perfluorinated sulfonimide…) allows the ability to control their properties and CO_2_ sorption in particular [4,10]. Among other tools for gaining the desirable control is the variation in the nature of polymer backbone and side groups as well as playing with macromolecular architecture by synthesis of linear, branched, star-shaped or cross-linked polymers. Moreover, it was recently shown that PILs possess several orders of magnitude higher CO_2_ sorption capability than respective ionic-liquid-like monomers and higher CO_2_ capture and desorption rates in comparison with ionic liquids (ILs) [12,13,14,15]. The light weight, the ease of handling, comparatively low cost and safety for humans and the environment were named among other advantages of PILs in the CO_2_ sorption process [4,10,12,16].

**Scheme 1:**
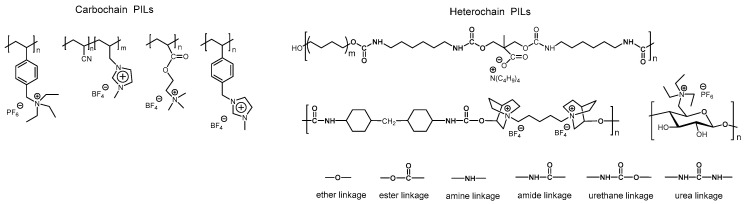
Examples of carbochain and heterochain PILs used for CO_2_ capture.

Over the past 10 years, various sorbents derived from both linear [4,17,18,19] and cross-linked [20,21,22] PILs have been investigated (see the examples in Scheme 1 and in Table 2). To enhance PILs’ CO_2_ sorption capacity, a number of approaches have been applied, such as PILs’ immobilization on carbon fibers [23,24], grafting of PILs on silica nanoparticles [25,26,27], incorporation of PILs into a metal–organic framework (MOF) [28,29,30,31] or preparation of ordered porous PIL-based crystallines [32]. It was found that the CO_2_ adsorption behavior of PILs is dependent on many factors, such as their chemical composition (nature of the cation and anion, type of the polymer backbone), molar mass and pore structure (surface area, pore size, atomic packing, etc.) [4,10].

Despite the fact that cross-linked PILs generally demonstrate higher sorption properties than their linear analogues, the study of the later is crucial for a more detailed fundamental insight into how the polymer structure affects the complex CO_2_ sorption mechanism [4,33]. Previously, the attention was mainly focused on understanding the influence of cations’ and anions’ structures on the CO_2_ sorption properties of PILs [4,13]. **Effect of the cation’s structure.** Comparing the influence of the cation’s structure on PILs’ CO_2_ capture, it was found that aliphatic cations (ammonium, quinuclidinium, etc.) commonly demonstrate higher CO_2_ sorption capacity than polyelectrolytes with aromatic cations (pyridinium, imidazolium, etc.) [16,17,34]. **Effect of the number of cations.** The higher the number of cations in the monomer unit (repeat unit charge density), the better the CO_2_ sorption ability of PILs [16]. **Effect of the anion’s structure.** The nature of the counter anion has a pronounced effect on the CO_2_ sorption of PILs as well. This was widely studied using such anions as R_1_COO (R_1_=CF_3_, C_3_F_7_, CH_3_), R_2_SO_3_ (R_2_=CF_3_, CH_3,_ C_6_H_4_-), (CF_3_SO_2_)_2_N, BF_4_, PF_6_, NO_3_, N(CN)_2_, B(CN)_4_, FeCl_3_Br, ZnCl_2_Br, CuCl_2_Br [14,15,16,18,19,27,35,36]. Among this vast variety of anions, the best CO_2_ uptake was demonstrated by CH_3_COO, B(CN)_4_, BF_4_ and PF_6_ containing PILs [15,16]. These results were in part explained for cellulose-based PILs by semi-empirical molecular dynamics simulations suggesting that different CO_2_ sorption capacities of PILs are driven by the cation–anion coordination peculiarities [15]. Comparing (CF_3_SO_2_)_2_N and PF_6_ anions, it was revealed that bulkier and significantly polar bis(trifluoromethylsulfonyl)imide ion is located near the most CO_2_-philic groups of imidazole cation and cellulose backbone, thus shielding them from interaction with CO_2_. In contrast, the same effect is much less pronounced for PF_6_ containing PILs, thus allowing for a higher number of centers for interaction with CO_2_ molecules and for higher CO_2_ sorption, respectively [15].

**Effect of the polymer backbone.** To date, the majority of PILs studied for CO_2_ capture are based on carbochain polymers, namely those derived from radical polymerization of styrene derivatives and (metha)acrylates (see Scheme 1 and Table 2) [4,10,14,17,37]. However, more detailed consideration allows for the conclusion that the polymer backbone plays an important role [4,10]. Thus, the subsequent transfer from carbochain to heterochain polymers is often accompanied by a significant increase in CO_2_ sorption [15,16,38,39]. This can be explained by the presence of polar groups in the polymer backbone capable of additional interaction with CO_2_ molecules—for example, by hydrogen bonding [4,10,40].

In ionic polyesters and polyethers, CO_2_ can additionally interact with the oxygen atom of the ester or ether linkages, as was shown by the dissolution study of various esters in supercritical scCO_2_ by Raman vibrational spectroscopy [41]. The secondary amine groups provide an effective adsorbate−adsorbent interaction in the capture of CO_2_ by polyamines [42]. In the same way, the amine group in ionic polyamides and polyurethanes [16,38,40] will offer an additional interaction with CO_2_ molecules and, as a result, increased CO_2_ capture capacity in comparison with carbochain PILs with similar cation/anion pairs. Therefore, the increase in the content of amine groups from two in ionic polyurethanes to four in ionic polyureas potentially should increase the points of interaction and subsequently the CO_2_ sorption capacity of PILs. Moreover, the synthesis of ionic polyureas with structures identical to previously developed ionic polyurethanes [16] will serve as a convenient model system for the comparison and estimation of the influence of hydrogen bonding on PILs’ CO_2_ capture.

**Scheme 2:**
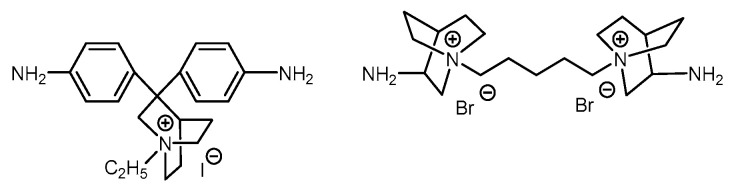
Structures of ionic diamines (monomers) used in the present study.

Utilization of PILs in the commercial CO_2_ capture process requires the elaboration of low-cost and simple ionic monomers. Thus, for the synthesis of ionic polyureas, two ionic diamines, namely 3,3-bis(4-aminophenyl)-1-ethylquinuclidin-1-ium iodide and 3-amino-1-(5-(3-aminoquinuclidin-1-ium-1-yl)pentyl)-quinuclidin-1-ium bromide, were suggested (Scheme 2). The first one was previously developed by our group [43], while the second is newly designed, taking into account the aim of the work consisting of the comparison of ionic polyureas with ionic polyurethanes reported antecedently [16]. Both monomers differ from known ionic diamines [44,45,46] by the simplicity of their synthesis, which consists of only two reaction steps (see Materials and Methods for details).

**Scheme 3:**
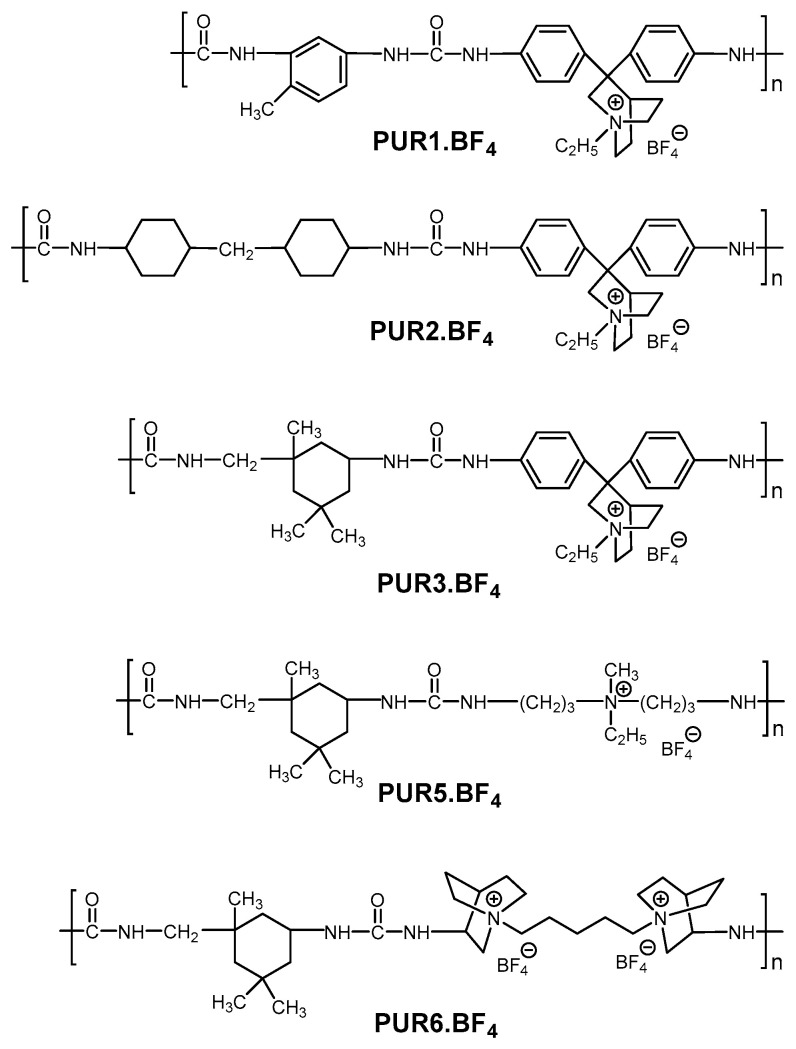
Structures of ionic polyureas (PURs) synthesized and studied in the present work.

Relying on the assumption that the increase in the number of secondary amino groups in the polymer backbone can improve the CO_2_ sorption properties of PILs, the aim of the present study was to synthesize a series of ionic polyureas and to investigate their ability for CO_2_ capture (Scheme 3). Thus, in this work, we report synthesis and properties investigation of five novel tetrafluoroborate PILs based on ionic polyureas (PURs) varying in the structure of diisocyanate (PUR1.BF_4_–PUR3.BF_4_), the nature of the cations (PUR3.BF_4_–PUR6.BF_4_) and their quantity (PUR3.BF_4_ and PUR6.BF_4_).

## 2. Materials and Methods

### 2.1. Materials

N-Methyldiethanolamine (>99%, Aldrich, Darmstadt, Germany), quinuclidinol-3 (99%, Aldrich, Darmstadt, Germany), 3-aminoquinuclidine dihydrochloride (98%, TCI Europe, Zwijndrecht, Belgium), potassium tetrafluoroborate (99%, Acros, Geel, Belgium), potassium carbonate (K_2_CO_3_, > 98%, Aldrich, Darmstadt, Germany), tin(II) 2-ethylhexanoate (98%, Aldrich, Darmstadt, Germany), iodoethane (99%, Aldrich, Darmstadt, Germany), N,N-dimethylformamide (DMF, anhydrous, 99.8%, Acros, Geel, Belgium), dichloromethane (CH_2_Cl_2_, anhydrous, 99.8%, Aldrich, Darmstadt, Germany), diethyl ether (Et_2_O, > 99%, Aldrich, Darmstadt, Germany), acetonitrile (CH_3_CN, anhydrous, 99.8%, Acros, Geel, Belgium), ethyl acetate (AcOEt, anhydrous, 99.8%, Acros, Geel, Belgium), 1,1,1,3,3,3-hexafluoro-propan-2-ol (HFIP, 99%, Apollo Scientific, Stockport, UK), 4,4′-methylene-bis(cyclohexyl isocyanate) (H_12_MDI, **2**, 99%, Covestro AG, Leverkusen, Germany) and isophorone diisocyanate (IPDI, **3**, 98%, Aldrich, Darmstadt, Germany) were used without purification.

Toluene-2,4-diisocyanate (TDI, **1**, 95%, Aldrich, Darmstadt, Germany) was purified by vacuum distillation. Meanwhile, 1,5-Dibromopentane (97%, Aldrich, Darmstadt, Germany) and N,N-bis-(3-aminopropyl)methylamine (**6**, 96%, Aldrich, Darmstadt, Germany) were purified by vacuum distillation over CaH_2_ and NaOH, respectively.

### 2.2. Synthesis of Ionic Diamine 3,3-Bis(4-Aminophenyl)-1-Ethylquinuclidin-1-Ium Iodide (4)

**Scheme 4:**
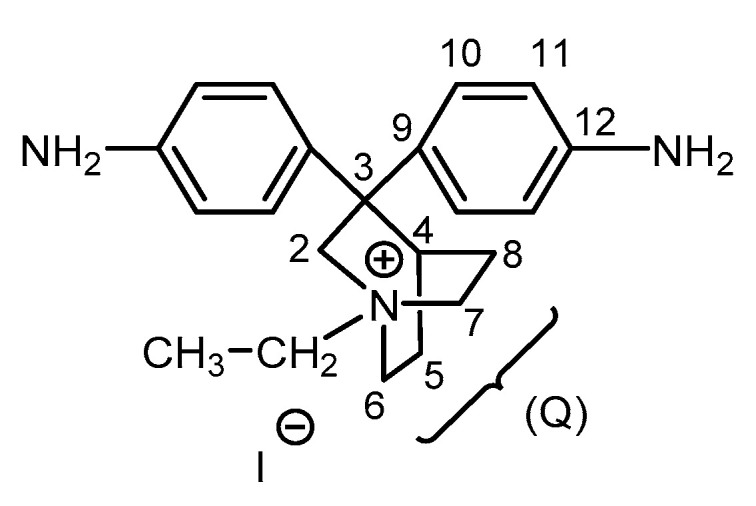
Structure of monomer **4** and its NMR assignment.

The 3,3-Bis(4-aminophenyl)-1-ethylquinuclidin-1-ium iodide monomer (**4**) was obtained according to the procedure reported by our group previously [43] (Scheme 4).

m.p. 293–294 °C; ^1^H NMR (400 MHz, DMSO-d6, δ ppm): 7.02–7.04 (d, 4H, J = 8.2 Hz, -C_6_H_4_-(10)), 6.46–6.48 (d, 4H, J = 8.2 Hz, -C_6_H_4_-(11)), 4.95 (s, 4H, -NH_2_), 4.07 (m, 2H, Q(2)), 3.10–3.36 (m, 7H, Q(4,6,7), N-C**H_2_**CH_3_), 1.77–1.88 (m, 4H, Q(5,8)), 1.30 (m, 3H, N-CH_2_C**H_3_**); ^13^C NMR (100 MHz, DMSO-d_6_, δ ppm): 145.5 (-C_6_H_4_-(12)), 137.4 (-C_6_H_4_-(9)), 126.9 (-C_6_H_4_-(10)), 113.9 (-C_6_H_4_-(11)), 59.4 (Q(2)), 49.2 (N-**C**H_2_CH_3_), 46.8 (Q(3,4)), 43.5 (Q(6,7)), 28.1 (Q(5)), 23.3 (q(8), N-CH_2_**C**H_3_); IR (KBr, ν, cm^−1^): 3466 (w, νNH), 3389 (m, νNH), 3037 (m, νCH_Ar_), 2980 (w, νCH_Alk_), 2875 (w, νCH_Alk_), 1619 (s), 1590 (s, νCC_Ar_), 1521 (s), 1465 (m, νCC_Ar_), 1445 (m, δ_as_CH_3_), 1410 (m), 1388 (m, δ_s_CH_3_), 1344 (w), 1260 (m), 1234 (m, νCC_Alk_), 1202 (m), 1111 (m, νCN), 1094 (w), 989 (m), 922 (m, νCN), 855 (w), 800 (w), 715 (s), 673 (s), 555 (w); Calc. for C_21_H_28_N_3_I (449.4): C 56.13; H 6.28; N 9.35; found: C 56.17; H 6.14; N 9.39.

### 2.3. Synthesis of 3-Amino-1-(5-(3-Aminoquinuclidin-1-Ium-1-Yl)Pentyl)-Quinuclidin-1-Ium Bromide (5)

**Scheme 5:**
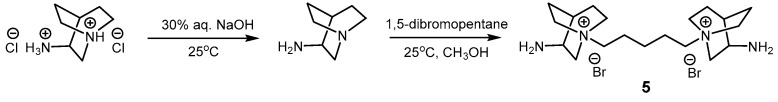
Synthesis of ionic monomer **5**.

Synthesis of ionic monomer **5** was conducted via two reaction steps, as presented in Scheme 5.

**Scheme 6:**
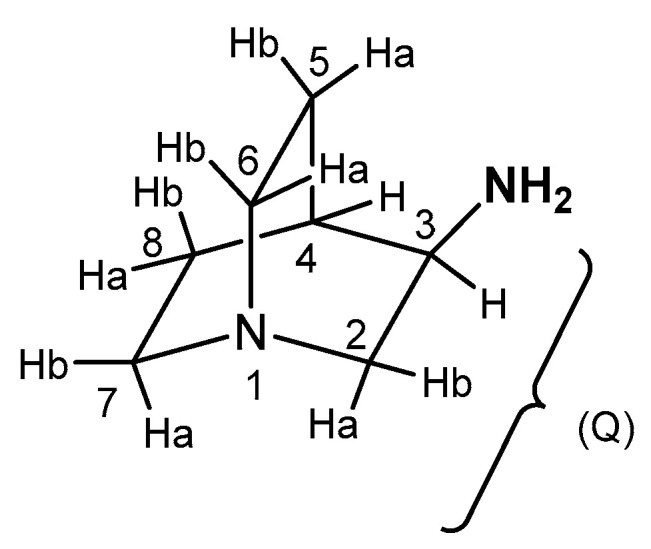
Structure of 3-aminoquinuclidine and its NMR assignment.

3-Aminoquinuclidine dihydrochloride (3.00 g, 0.015 mol) was dissolved in 30% NaOH aqueous solution at room temperature. The solution was further extracted with diethyl ether (5 × 25 mL) and the combined extracts were dried over K_2_CO_3_. Potash was filtered off and diethyl ether was evaporated under reduced pressure. Then, 3-Aminoquinuclidine (Scheme 6) was obtained as white crystalline solid and was dried for 8 h at 40 °C/12 mm Hg. Yield: 1.23 g (64%); m.p. = 219–222 °C (218–220 °C [47]);

^1^H NMR (400 MHz, DMSO-d6, δ ppm): 2.93 (m, 1H, Q (2a)), 2.79 (m, 1H, Q (3)), 2.72–2.30 (m, 6H, Q (6,7), NH2), 2.14 (m, 1H, Q (2b)) 1.84 (m, 1H, Q (5a)), 1.53 (m, 1H, Q (8b)), 1.48 (m, 1H, Q (4)), 1.39 (m, 1H, Q (8a)), 1.18 (m, 1H, Q (5b)). ^13^C NMR (100 MHz, DMSO-d_6_, δ ppm): 57.20 (Q(2)), 47.25 (Q(3)), 46.67 (Q(6)), 45.44 (Q(7)), 28.23 (Q(4)), 25.45 (Q(8)), 18.43 (Q(5)).

The solution of 1,5-dibromopentane (1.30 g, 5.67 mmol) in 20 mL of anhydrous CH_3_OH was added dropwise to the solution of 3-aminoquinuclidine (1.50 g4, 11.91 mmol) in 30 mL of CH_3_OH at room temperature under inert atmosphere. The reaction mixture was stirred for 24 h at ambient temperature, whereupon product was isolated by precipitation into the excess of anhydrous Et_2_O and subsequent filtration. Diamine **5** was obtained as white powder that was dried for 8 h at 60 °C/1 mmHg. Yield: 2.50 g (93%); m.p. 89-90°C; ^1^H NMR (400 MHz, DMSO-d_6_, δ ppm): 4.0–3.7 (br. m., 4H, NH2) 3.66 (m, 2H, Q (2a)), 3.39 (br. m, 10H, Q (3,6,7)), 3.22 (br. t, 4H, N-**CH_2_**-CH_2_-CH_2_, J = 7.3 Hz), 2.98 (m, 2H, Q (2b)), 1.98 (m, 2H, Q (4)), 1.89–1.60 (m, 12H, Q (5,8), N-CH_2_-**CH_2_**-CH_2_), 1.22–1.24 (m, 2H, N-CH_2_-CH_2_-**CH_2_**-); ^13^C NMR (100 MHz, DMSO-d_6_, δ ppm): 67.30 (Q (2)), 63.88 (Q(3)), 62.94 (N-**CH_2_**-CH_2_-CH_2_), 53.29 (Q(6, 7), 29.80 (Q(4)), 26.51 (Q(5 or 8)), 24.35 (N-CH_2_-CH_2_-**CH_2_**), 21.08 (N-CH_2_-**CH_2_**-CH_2_), 17.83 (Q(5 or 8)); Calc. for C_19_H_38_N_4_Br_2_ (482.3): C 47.31; H 7.94; N 11.62; found: C 47.06; H 7.99; N 11.45.

### 2.4. Polycondensation

Iodide PURs, namely **PUR1.I–PUR3.I**, were synthesized by polycondensation of respective diisocyanates with ionic diamine **4**. Bromide **PUR6.Br** and neutral **PUR4** were prepared by reaction of IPDI diisocyanate with ionic diamine **5** and noncharged diamine **6**, respectively. All mentioned polyureas were synthesized following the general procedure reported for **PUR1.I** below, with the exception that **PUR1.I–PUR3.I** were precipitated from DMF solution into the excess of water, while **PUR6.Br** and **PUR4** were precipitated into the acetone excess.

Ionic diamine **4** (1.0370 g, 2.3 mmol) and diisocyanate **1** (0.4019 g, 2.3 mmol) were dissolved in 7 mL of anhydrous DMF in a Schlenk flask inside an argon-filled glovebox (MBRAUN MB-Labstar, Garching, Germany, H_2_O and O_2_ content < 0.5 ppm). The flask was closed with a rubber septum, taken out of the glovebox and placed in a preheated to 60 °C oil bath.

Then, 2-(Ethyl)hexanoate tin (II) (0.0233 g, 0.06 mmol, 2.5% mol with respect to **4**) was dissolved separately in 0.5 mL of anhydrous DMF inside the glovebox. The flask was then taken out of the glovebox and the catalyst solution was injected via syringe technique into the preheated solution of monomers. The reaction mixture was stirred at 60 °C for 15 h, whereupon polymer was isolated by precipitation into the excess of water, collected by centrifugation (15,000 rpm, 10 min, 5 °C), and thoroughly washed with water and acetone. **PUR1.I** was isolated as yellow powder and dried for 12 h at 100 °C/1 mm Hg. Yield: 1.24 g (87%).

### 2.5. Quaternization of PUR.4

The solution of C_2_H_5_I (4.67 g, 29.93 mmol) in 20 mL of DMF was added dropwise to the solution of **PUR4** (1.10 g, 2.99 mmol) in 50 mL of DMF preheated at 40 °C. Stirring was continued at 40 °C for 12 h, whereupon polymer was isolated by precipitation into the excess of ethyl acetate and thoroughly washed with ethyl acetate. **PUR5.I** in a form of white powder was dried for 12 h at 70 °C/1 mm Hg. Yield: 1.48 g (95%).

### 2.6. Ion Exchange

All ionic PURs with tetrafluoroborate anions, namely **PUR1.BF_4_–PUR6.BF_4_**, were synthesized via anion metathesis reaction with the excess of KBF_4_. In the case of hydrophobic **PUR1.I–PUR3.I**, the ion exchange reaction was performed in DMF:CH_3_CN mixture (4:1 by volume), while for hydrophilic **PUR5.I** and **PUR6.Br**, metathesis was carried out in water. General procedures are reported below for **PUR1.BF_4_** and **PUR6.BF_4_**.

KBF_4_ (0.48 g, 3.82 mmol) was added in one shot to the solution of **PUR1.I** (1.59 g, 2.55 mmol) in 50 mL of DMF:CH_3_CN (4:1 by volume). The reaction suspension was stirred at RT for 12 h. The desired polymer was isolated by precipitation into the excess of water, thoroughly washed with water and dried for 4 h at ambient temperature. Further on, the polymer was redissolved in HFIP and precipitated into the excess of ethyl acetate. **PUR1.BF_4_** in the form of yellow-beige powder was dried for 12 h at 100 °C/1 mm Hg. Yield: 1.37 g (92%).

The solution of KBF_4_ (1.12 g, 8.87 mmol) in 10 mL of water was added dropwise to the solution of **PUR6.Br** (2.50 g, 3.55 mmol) in 45 mL of water. The precipitation of the polymer was immediately observed, whereupon it was collected by centrifugation (15,000 rpm, 10 min, 5 °C), thoroughly washed with water and dried for 4 h at ambient temperature. Afterwards, polymer was redissolved in HFIP and precipitated into the excess of ethyl acetate. **PUR6.BF_4_** in the form of yellow powder was dried for 12 h at 160 °C/1 mm Hg. Yield: 1.71 g (67%).

**PUR1.BF_4_**

**Scheme 7:**
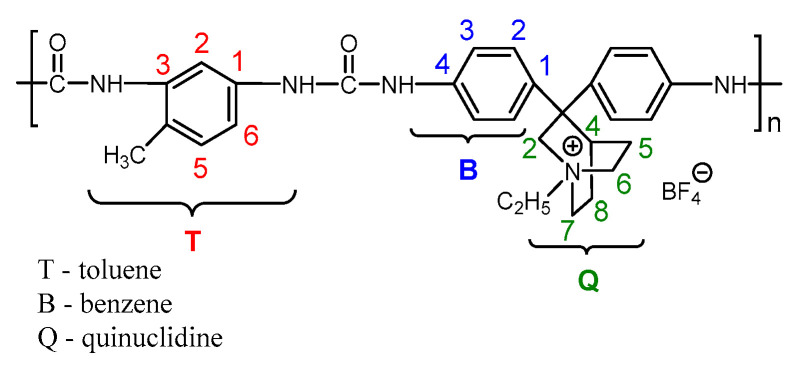
Structure of **PUR1.BF_4_** and its NMR assignment.

^1^H NMR (600 MHz, DMSO-d_6_, δ ppm, Scheme 7): 9.04, 8.68, 8.57 (m, 4H, NH), 7.90 (m, 1H, T-2), 7.39 (m, 8H, B-2, B-3), 7.22 (m, 1H, T-6), 7.07 (m, 1H, T-5), 4.20 (m, 2H, Q-2), 3.43 (m, 2H, NC**H**_2_CH_3_), 3.33 & 3.14 (m, 4H, Q-6 & Q-7), 3.32 (m, 1H, Q-4), 2.18 (s, 3H, C**H**_3_), 1.94 & 1.77 (m, 4H, Q-5 and Q-8), 1.33 (m, 3H, NCH_2_C**H**_3_); ^13^C NMR (151 MHz, DMSO-d_6_, δ ppm): 152.43 (**C**ONH), 138.84 (B-1,4), 137.47 (T-3), 130.79 (T-5), 126.94 (B-2,6), 120.59 (T-4), 118.69 (B-3,5), 113.15 (T-6), 111.33 (T-2), 62.50 (Q-2), 59.14 (N**C**H_2_CH_3_), 52.97 (Q-6, Q-7), 45.95 (Q-3), 27.04 (Q-4), 21.12 (Q-5, Q-8), 17.83 (**C**H_3_), 7.98 (NCH_2_**C**H_3_); IR, (ATR mode, ν, cm^−1^): 3645 (w), 3625 (w), 3392 (w, νNH), 3250 (w, νNH), 1689 (s, νC=O), 1595 (s), 1512 (vs, νC=O), 1409 (s, νCN), 1409 (m), 1320 (m), 1293 (m), 1210 (vs), 1127 (w), 1049 (vs, νBF), 741 (m), 664 (w), 640 (w), 598 (w), 556 (vs), 530 (m); M_w_ = 12,000 g/mol; M_w_/M_n_ = 1.42 (GPC); T_g_ = 286 °C (TMA).

**PUR2.BF_4_**

**Scheme 8:**
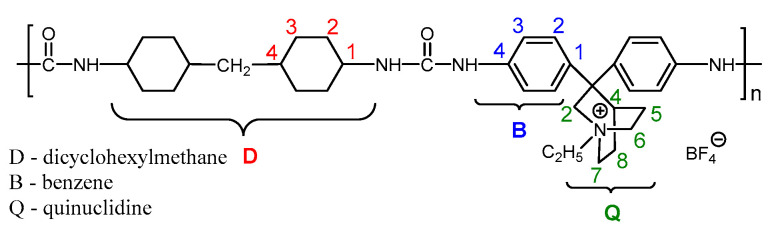
Structure of **PUR2.BF_4_** and its NMR assignment.

^1^H NMR (600 MHz, DMSO-d_6_, δ ppm, Scheme 8): 8.31 (m, 2H, NH), 7.28 (m, 8H, B-2, B-3), 6.26–5.49 (m, 2H, NH), 4.16 (m, 2H, Q-2), 3.40 (m, 2H, NC**H**_2_CH_3_), 3.31 & 3.13 (m, 4H, Q-6 & Q-7), 3.31 (m, 1H, Q-4), 1.95 & 1.75 (m, 4H, Q-5 & Q-8), 1.32 (m, 3H, NCH_2_C**H**_3_). 1.82–0.90 (br.m., 20H, CH-C**H**_2_-CH, D-2, D-3, D-4); ^13^C NMR (151 MHz, DMSO-d_6_, δ ppm): 154.33 (**C**ONH), 138.24 (B-1,4), 126.22 (B-2,6), 117.54 (B-3,5), 61.85 (Q-2), 58.52 (N**C**H_2_CH_3_), 52.44 (Q-6, Q-7), 45.04 (Q-3), 48.03 & 44.00 (D-1), 26.53 (Q-4), 20.58 (Q-5, Q-8), 7.33 (NCH_2_**C**H_3_). 32.91 & 32.21 (D-4), 32.66 (D), 31.50 (D), 29.46 (CH-C**H**_2_-CH), 27.29 (D); ^19^F NMR (565 MHz, DMSO-d_6_, δ ppm): -148.2; IR, (ATR mode, ν, cm^−1^): 3645 (w), 3427 (w, νNH), 3332 (w, νNH), 2922 (m, νCH_Alk_), 2852 (m, νCH_Alk_), 1675 (s, νC=O), 1595 (m), 1513 (vs, νC=O), 1451 (m, νCN), 1407 (m), 1323 (s), 1197 (s), 1048 (vs, νBF), 740 (w), 713 (w), 664 (w), 643 (w), 598 (w), 556 (s), 509 (m); M_w_ = 15,800 g/mol; M_w_/M_n_ = 1.61 (GPC); T_g_ = 271 °C (TMA).

**PUR3.BF_4_**

**Scheme 9:**
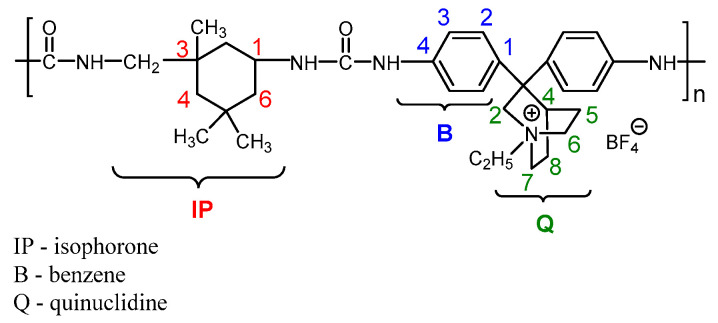
Structure of **PUR3.BF_4_** and its NMR assignment.

^1^H NMR (600 MHz, DMSO-d_6_, δ ppm, Scheme 9): 8.95 & 8.48–8.30 (br m, 2H, NH), 7.28 (m, 8H, CH(Ar)), 6.2–5.5 (br m., 2H, NH), 4.14 (m, 2H, Q-2), 3.76 (m, 1H, IP-1), 3.40 (m, 2H, NC**H**_2_CH_3_), 3.28 (m, 3H, Q-4, Q-6 & Q-7), 3.12 (m, 2H, Q-6 & Q-7), 2.88 & 2.79 (m, 2H, C**H**_2_NHCO), 1.91 & 1.78 (m, 4H, Q-5 and Q-8), 1.57 (m, 1H, IP-6), 1.52 (m, 1H, IP-2), 1.31 (m, 3H, NCH_2_C**H**_3_), 1.13 & 1.02 (m, 2H, IP-4), 1.00 (s, 3H, CH_3_ at IP-5), 0.96 (s, 2.25H, CH_3_ at trans-IP-3), 0.89 (s, 4H, IP-6 & CH_3_ at IP-5), 0.85 (m, 1H, IP-2), 0.82 (s, 0.75H, cis-IP-3); ^13^C NMR (151 MHz, DMSO-d_6_, δ ppm): 155.33 & 154.41 (**C**ONH), 138.46–138.15 (B-1,4), 126.16 (B-2,6), 117.34 (B-3,5), 61.91 (Q-2), 58.34 (N**C**H_2_CH_3_), 52.54 (**C**H_2_NHCO), 52.23 (Q-6, Q-7), 46.65 (IP-4), 46.02 (IP-6), 45.44 (Q-3), 42.15 (IP-1), 41.96 (IP-2), 35.99 (IP-3), 34.74 (CH_3_ at IP-5), 31.33 (IP-5), 29.63 (CH_3_ at cis-IP-3), 27.28 (CH_3_ at IP-5), 26.29 (Q-4), 23.01 (CH_3_ at trans-IP-3), 20.40 (Q-5, Q-8), 7.16 (NCH_2_**C**H_3_); IR, (ATR mode, ν, cm^−1^): 3648 (w), 3422 (w, νNH), 3339 (w, νNH), 2951 (w, νCH_Alk_), 2904 (w, νCH_Alk_), 1681 (s, νC=O), 1596 (s), 1513 (vs, νC=O), 1464 (m, νCN), 1408 (m), 1321 (m), 1226 (s), 1197 (s), 1050 (vs, νBF), 740 (w), 698 (w), 645 (w), 601 (w), 556 (vs), 506 (m); M_w_ = 101,500 g/mol; M_w_/M_n_ = 4.12 (GPC); T_g_ = 276 °C (TMA).

**PUR5.BF_4_**

**Scheme 10:**
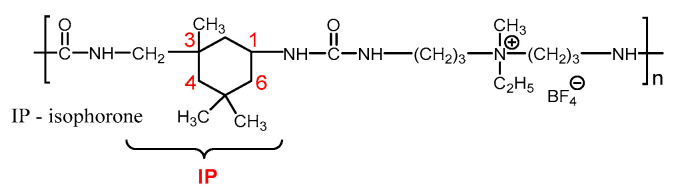
Structure of **PUR5.BF_4_** and its NMR assignment.

^1^H NMR (600 MHz, DMSO-d_6_, δ ppm, Scheme 10): 6.3–5.2 (br m., 4H, NH), 3.69 (m, 1H, IP-1), 3.40 (m, 2H, NC**H**_2_CH_3_), 3.18 (m, 4H, NHCH_2_CH_2_C**H**_2_), 3.05 (s, 3H, NC**H**_3_), 3.00 (m, 4H, NHC**H**_2_CH_2_CH_2_), 2.77 & 2.71 (m, 2H, C**H**_2_NHCO), 1.51 (m, 1H, IP-6), 1.47 (m, 4H, NHCH_2_C**H**_2_CH_2_), 1.46 (m, 1H, IP-2), 1.20 (m, 3H, NCH_2_C**H**_3_), 1.09 (m, 1H, IP-4), 0.98 (s, 3H, CH_3_ at IP-5), 0.95 (m, 1H, IP-4) 0.92 (s, 2.25H, CH_3_ at trans-IP-3) 0.87 (s, 3H, CH_3_ at IP-5), 0.82 (m, 1H, IP-6), 0.76 (s, 0.75H, cis-IP-3). 0.75 (m, 1H, IP-2); ^13^C NMR (151 MHz, DMSO-d_6_, δ ppm): 158.40 & 157.40 (**C**ONH), 58.26 (NHCH_2_CH_2_**C**H_2_), 56.24 (m, 2H, NC**H**_2_CH_3_), 52.97 (**C**H_2_NHCO), 47.14 (s, 3H, NC**H**_3_), 46.75 (IP-4), 46.28 (IP-6), 42.94 (IP-1), 42.08 (IP-2), 36.04 (IP-3), 34.96 (NH**C**H_2_CH_2_CH_2_), 34.72 (CH_3_ at IP-5), 31.40 (IP-5), 29.53 (CH_3_ at cis-IP-3), 27.27 (CH_3_ at IP-5), 23.20 (NHCH_2_**C**H_2_CH_2_), 22.90 (CH_3_ at trans-IP-3), 7.39 (NCH_2_C**H**_3_); ^19^F NMR (565 MHz, DMSO-d_6_, δ ppm): -148.1; IR, (ATR mode, ν, cm^−1^): 3291 (w, νNH), 3139 (w, νCH_Alk_), 1637 (s, νC=O), 1563 (vs, νC=O), 1478 (m, νCN), 1436 (m), 1399 (w), 1305 (w), 1258 (s), 1024 (vs, νBF), 772 (w), 661 (w), 630 (w), 590 (w), 521 (m); M_w_ = 173,500 g/mol; M_w_/M_n_ = 2.32 (GPC); T_g_ = 153 °C (TMA).

**PUR6.BF_4_**

**Scheme 11:**
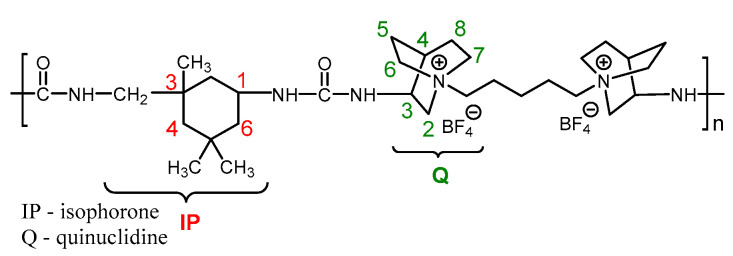
Structure of **PUR6.BF_4_** and its NMR assignment.

^1^H NMR (600 MHz, DMSO-d_6_, δ ppm, Scheme 11): 7.8–5.7 (br m., 4H, NH), 3.96 (m, 2H, Q-3), 3.72 (m, 3H, Q-2 and IP-1), 3.34 (m, 8H, Q-6 & Q-7), 3.15 (m, 4H, NC**H**_2_CH_2_CH_2_), 3.01 (m, 2H, Q-2), 2.84 & 2.71 (m, 2H, C**H**_2_NHCO), 2.02 (m, 4H, Q-4 and Q-5), 1.92 (m, 4H, Q-8), 1.84 (m, 2H, Q-5), 1.67 (m, 4H, NCH_2_C**H**_2_CH_2_), 1.53 (m, 1H, IP-6), 1.45 (m, 1H, IP-2), 1.24 (m, 2H, NCH_2_CH_2_C**H**_2_), 1.12 (m, 1H, IP-4), 0.99 (s, 2.25H, CH_3_ at trans-IP-5), 0.98 (m, 1H, IP-4) 0.96 (s, 0.75H, cis-IP-5), 0.93 (s, 3H, CH_3_ at trans-IP-3 & cis-IP-5), 0.88 (s, 2.25H, CH_3_ at trans-IP-5), 0.86 (m, 1H, IP-6), 0.79 (s, 0.75H, cis-IP-3), 0.79 (m, 1H, IP-2); ^13^C NMR (151 MHz, DMSO-d_6_, δ ppm): 157.89 & 156.96 (**C**ONH), 62.37 (N**C**H_2_CH_2_CH_2_), 61.19 (Q-2), 53.08 (**C**H_2_NHCO), 52.75 (Q-6, Q-7), 46.53 (IP-4), 46.13 (IP-6), 44.58 (Q-3), 42.16 (IP-1), 41.96 (IP-2), 35.91 (IP-3), 34.77 (CH_3_ at trans-IP-5), 34.51 (CH_3_ at cis-IP-5), 31.32 (IP-5), 29.80 (CH_3_ at cis-IP-3), 27.23 (CH_3_ at trans-IP-5), 26.63 (CH_3_ at cis-IP-5), 24.24 (Q-4), 22.07 (Q-8), 18.03 (Q-5), 20.54 (NCH_2_**C**H_2_CH_2_), 22.70 (NCH_2_CH_2_**C**H_2_), 22.98 (CH_3_ at trans-IP-3); ^19^F NMR (565 MHz, DMSO-d_6_, δ ppm): -148.2; IR, (ATR mode, ν, cm^−1^): 3604 (w), 3565 (w), 3409 (w, νNH), 3308 (w, νNH), 2952 (m, νCH_Alk_), 1650 (s, νC=O), 1549 (vs, νC=O), 1492 (m), 1466 (m, νCN), 1387 (m), 1366 (m), 1307 (m), 1286 (m), 1246 (m), 1190 (m), 1052 (vs, νBF), 898 (m), 833 (m), 733 (m), 649 (s), 585 (s); M_w_ = 39,000 g/mol; M_w_/M_n_ = 1.97 (GPC); T_g_ = 209 °C (TMA).

### 2.7. Methods

NMR spectra were recorded on AMX-400 and Avance III HD 600 MHz spectrometers (Bruker, Billerica, MA, USA) at 25 °C in the indicated deuterated solvents and are listed in ppm. The signal corresponding to the residual protons of the deuterated solvent was used as an internal standard for ^1^H and ^13^C NMR, while the C_6_F_6_ was utilized as an external standard for ^19^F. Signal assignment was performed using 2D NMR techniques: heteronuclear single quantum coherence (HSQC), heteronuclear multiple bond correlation (HMBC), H-H correlation spectroscopy (H-H COSY). The following abbreviations were used in the spectra description in order to refer to the fragments: dicyclohexylmethane (D), benzene (B), toluene (T), isophorone (IP) and quinuclidine (Q). IR spectra were acquired on a Nicolet Magna-750 Fourier IR-spectrometer using KBr pellets or on Brucker Tensor 27 Fourier IR-spectrometer (Bruker, Billerica, MA, USA) using ATR technology (128 scans, resolution is 2 cm^−1^) and Spectragryph optical spectroscopy software [48].

A 1200 Infinity gel permeation chromatograph (GPC, Agilent Technologies, Santa Clara, CA, USA) was used to determine M_n_, M_w_ and M_w_/M_n_ of the ionic polyureas. The chromatograph was equipped with an integrated IR detector, a PL PolarGel-M column and a PL PolarGel-M guard column (Agilent Technologies, Santa Clara, CA, USA). The 0.1 M solution of NH_4_BF_4_ in DMF was used as an eluent, the flow rate was maintained at 1.0 mL/min and the measurements were performed at 50 °C. Polystyrene standards (EasiVial PS-M, Agilent Technologies, Santa Clara, CA, USA, M_p_ = 162–500 × 10^3^) were used to perform calibration.

Thermal gravimetric analysis (TGA) was carried out in air and under inert atmosphere (N_2_) on a TGA2 STARe System (Mettler Toledo, Greifensee, Zwitzerland), applying a heating rate of 5 °C/min. Thermal mechanical analysis (TMA) of PURs was performed under inert atmosphere (Ar) using a DIL 402C dilatometer (NETZSCH, Selb, Germany) at a heating rate of 5 °C/min and a constant load of 0.08 MPa. PUR samples were hermetically sealed in aluminum pans inside the argon-filled glovebox (MBRAUN MB-Labstar, Garching, Germany, H_2_O and O_2_ content < 0.5 ppm).

The CO_2_ adsorption isotherms of the synthetized PILs were determined at 0 °C in a Nova 4200 volumetric apparatus (Quantachrome, now Anton Paar, Graz, Austria) in accordance with the standard procedures established by Quantachrome for measuring CO_2_ adsorption capacities at 0 °C in porous materials. Around 250 mg of PILs sample in powder form (all ionic PURs in this work were precipitated from the diluted solutions as powders with low particle size (see vide supra)) were introduced in the cell consisting of a glass tube with a bulb and were degassed at 80 °C for 18 h under vacuum prior to measurement in order to eliminate the sample humidity and any other adsorbed gases. For the selected PIL samples, CO_2_ adsorption and desorption cycles were carried out and five correlative isotherms were done without degasification step between the measurements. The 25 adsorption points (and 22 desorption points when it was measured) were selected in the pressure range from 0.006 to 1 bar, corresponding to a relative pressure range from 0.0002 to 0.03 (note: P^0^ for CO_2_ at 0 °C is 34.85 bar). The equilibrium time (both for the adsorption and desorption) was 300 s. A non-ideality factor of 8.93 × 10^−6^ mmHg^−1^, obtained using the Helmholtz equation-of-state proposed by Span and Wagner [49] and recommended by the National Institute of Standards and Technologies of USA (NIST), was used for determining the real CO_2_ density.

Cycling in thermobalance. Several cycles under an alternative CO_2_ or N_2_ flow at room temperature were evaluated in a thermogravimetric analyser (CI Electronics microbalance, now CI Precision, Salisbury, UK). All experiments were carried out at atmospheric pressure and using a gas flow rate (both for CO_2_ and N_2_) of 50 mL/min. **PUR1.BF_4_** or **PU1.BF_4_** samples (about 70 mg) were placed in the quartz cap and the weight was recorded at regular intervals (6 s). The samples were heated at 80 °C for 4 h under a N_2_ flow of 50 mL/min to remove any adsorbed gas (i.e., to degas the samples). The temperature was controlled at 25 °C using a thermostatic bath and then the gas was changed to CO_2_ and kept under this CO_2_ stream for 1 h (in this time, saturation and a constant weight were reached). The gas was switched back to N_2_ and held for 2 or 3 h. In this time, the complete desorption of the CO_2_ was not achieved since the initial weight was not recovered. At the end of the third cycle, the samples were kept under current of N_2_ for 8 h to achieve the initial weight (i.e., complete desorption of CO_2_ was reached) and the other two cycles were repeated.

## 3. Results

### 3.1. Synthesis of Ionic Diamines

Previous investigation performed by our group on CO_2_ sorption of PILs demonstrated the advantage of quinuclidinium and diquinuclidinium cations over ammonium and imidazolium ones [16]. Keeping this in mind, the work started with the design of ionic diamines. Mono quinuclidinium diamine **4**, namely 3,3-bis(4-aminophenyl)-1-ethylquinuclidin-1-ium iodide, was prepared in accordance with the procedure reported by our group previously [43]. As for the diquinuclidinium monomer **5**, the reaction pathway consisting of two steps was developed (Scheme 4). The first step consisted of treatment of 3-aminoquinuclidine dihydrochloride with 30% NaOH aqueous solution and subsequent extraction with diethyl ether to give 3-aminoquinuclidine. The double excess of the latter in a second step was quarternized by 1,5-dibromopentane, applying mild conditions (Scheme 4). It should be mentioned that the slight excess of 3-aminoquinuclidine is required to produce difunctional monomer. Afterwards, the precipitation from methanol solution into the excess of diethyl ether results in purification of monomer **5** and its isolation in 93% yield.

The structure and purity of ionic diamines **4** and **5** were proven by ^1^H and ^13^C NMR spectroscopy as well as by elemental analysis. Diamine **4** represented beige-yellow crystalline solid, while **5** was isolated as white crystalline powder. The melting points determined for **4** and **5** were equal to 293–294 and >360 °C, respectively.

### 3.2. Synthesis of Ionic Polyureas

**Scheme 12:**
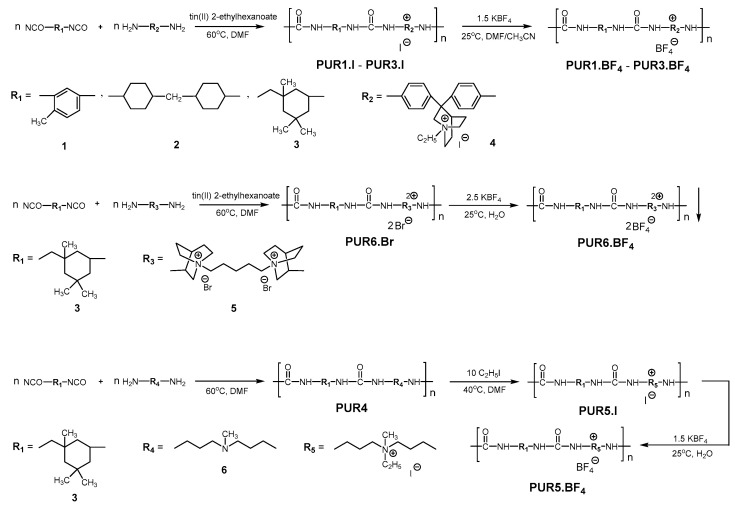
Synthetic pathways for the preparation of ionic polyureas **PUR1.BF_4_**–**PUR3.BF_4_** and **PUR5.BF_4_**–**PUR6.BF_4_**.

A series of ionic polyureas were synthesized by combining ionic monomers **4** and **5** with three commercial diisocyanates, as shown in Scheme 12. The optimal conditions (4 mol/L solution in DMF, 2.5 mol of catalyst 2-(ethyl)hexanoate tin (II) with respect to diamine, 60 °C for 15 h) determined previously for polycondensation of diisocyanates with ionic diols [16] were applied for the synthesis of **PUR1.I**–**PUR3.I**, **PUR4** and **PUR6.Br** (Scheme 12). The reaction was rather slow for aromatic ionic diamine **4**, while for aliphatic **6**, it proceeded faster and required no catalyst addition. The resultant polymers were isolated in 86–89% yield.

Synthesis of tetrafluoroborate polyureas, namely **PUR1.BF_4_–PUR6.BF_4_**, was carried out via anion metathesis reaction with the excess of KBF_4_. In the case of hydrophobic **PUR1.I–PUR3.I**, the ion exchange reaction was performed in DMF:CH_3_CN mixture, while for hydrophilic **PUR5.I** and **PUR6.Br**, metathesis was carried out in water. The isolated yields ranged from 67 to 96%. The structure, composition and purity of **PUR1.BF_4_–PUR6.BF_4_** were supported by ^1^H, ^13^C and ^19^F NMR and FTIR spectroscopy (see Figures S1–S2 in the Supplementary Materials for most complex **PUR6.BF_4_**). ^19^F NMR shows a singlet at −148. 2 ppm assigned to BF_4_ anion (Figure S1c). FTIR spectrum of **PUR6.BF_4_** presents the characteristic vibration bands of polyurea, as depicted in Figure S2. The broad band at 3400–3300 cm^−1^ is related to NH stretching. The peaks at 2952 and 2916 cm^−1^ are associated with aliphatic CH stretching. The strong bands at 1650 cm^−1^ (amide I, stretching of carbonyl group) and 1551 cm^−1^ (amide II, NH bending) are assigned to the urea linkage. The strong band at 1052 cm^−1^ relates to the BF_4_ anion. The complete conversion of the NCO groups was proven by the absence of the bands at 2250–2270 cm^−1^.

### 3.3. Properties of Ionic Polyureas

#### 3.3.1. Solubility and Molecular Weights

**PUR1.BF_4_–PUR6.BF_4_** are amorphous polymers and were found to be soluble in polar aprotic solvents (i.e., DMF, DMSO, DMAc and NMP) and HFIP. Probably due to the hydrogen bonding, they were not soluble in acetone or acetonitrile, in contrast to structurally similar ionic PUs [16]. The same relates to methanol, ethyl acetate and diethyl ether, where the ionic PURs were not soluble either. Finally, tetrafluoroborate anion imparted hydrophobic properties to all obtained PURs.

The molar masses of ionic PURs were estimated by size exclusion chromatography (SEC). To suppress the insufficiently charged screening or the so-called “polyelectrolyte effect” during SEC studies, an electrolyte, namely NH_4_BF_4_, was added to the polymer solution in DMF. Polycondensation of ionic monomers **4** and **5** resulted in the formation of high molar mass polyureas with weight average molar masses (M_w_) ranging from 12 to 174 kg/mol for **PUR1.BF_4_–PUR6.BF_4_** (Table 1).

#### 3.3.2. Thermal Properties

Thermal properties of ionic **PUR1.BF_4_–PUR6.BF_4_** were assessed via mechanical (TMA) and thermogravimetric analyses (TGA). TMA of polyelectrolytes performed in inert atmosphere revealed the following evolution of glass transition temperatures:

T_g_**PUR1.BF_4_** (286 °C) > T_g_**PUR3.BF_4_** (276 °C) ≈ T_g_**PUR2.BF_4_** (271 °C) > T_g_**PUR6.BF_4_** (209 °C) > T_g_**PUR5.BF_4_** (153 °C)

It can be concluded that **PUR1.BF_4_** based on aromatic polymers demonstrated the highest T_g_ among studied polyureas. The change in aromatic toluene-2,4-diisocyanate (**1**) to aliphatic isophorone diisocyanate (**3**) and 4′-methylene-bis(cyclohexyl isocyanate) (**2**) monomers resulted in the decrease in polymers T_g_ (Table 1, entries 2–3). Further transfer to completely aliphatic polymers **PUR5.BF_4_**–**PUR6.BF_4_** allowed us to significantly decrease polyelectrolytes’ heat resistance (Table 1, entries 4–5). At this, **PUR6.BF_4_** with cycloaliphatic quinuclidinium cation showed higher T_g_ values in comparison with **PUR5.BF_4_** based on quaternary ammonium (Table 1, entries 4–5).

**Figure 1:**
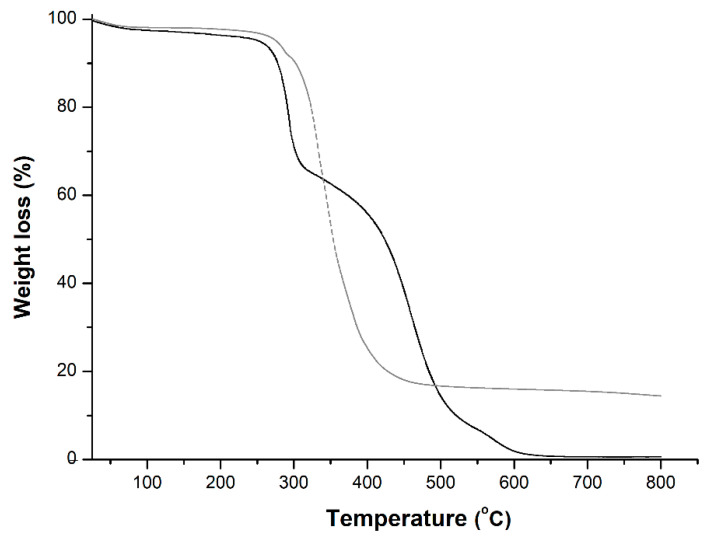
TGA traces of **PUR1.BF4** under air (solid line) and inert atmosphere (dash line).

Thermal stability of PILs was performed under air and under inert atmosphere (Figure 1 and Table 1). PUR temperatures of onset weight loss (T_onset_) on air ranged from 195 to 265 °C (Table 1). The weight loss profiles of **PUR1.BF_4_**–**PUR6.BF_4_** on air revealed a two-step degradation mechanism (Figure 3). The first weight loss step took place between 240 and 360 °C and can be probably attributed to the degradation of aliphatic quinuclidinium cation. The second step occurred over 400 °C (Figure 1). In contrast, the TGA analysis performed under inert atmosphere showed a one-step degradation mechanism. However, the T_onset_ values practically coincided with those determined by TGA on air (Table 1).

#### 3.3.3. CO_2_ Sorption

**Figure 2:**
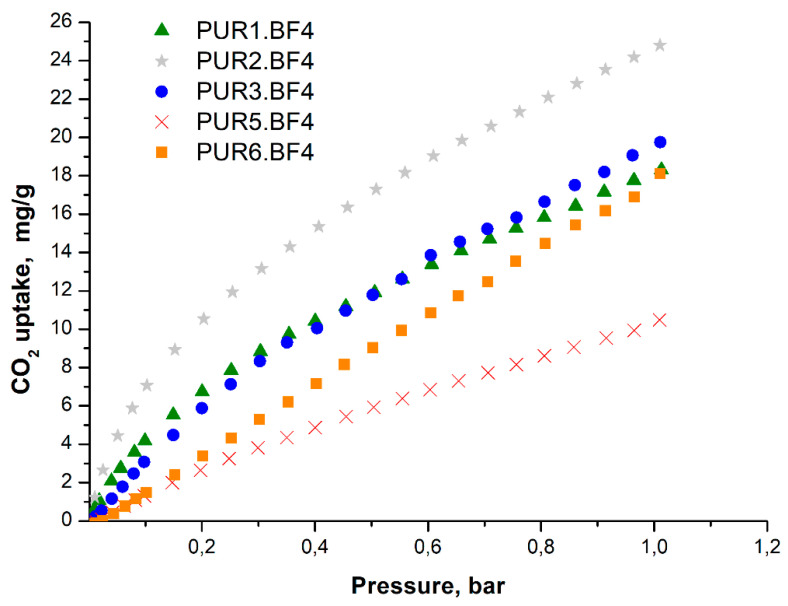
CO_2_ gas absorption isotherms of ionic PURs at 0 °C.

Experimental results of CO_2_ sorption for all synthesized PILs are shown in Figure 2 and Table 1. As CO_2_ adsorption is temperature dependent, the measurements were performed at 0 °C as it is a standard temperature for porosity studies of solids with CO_2_ as adsorbate as well as for characterization of materials with narrow microporosity [4,10,50,51,52]. The variation in the comonomers’ structure in five synthesized ionic polyureas allowed us to investigate the effect of the isocyanate’s nature (aromatic/cycloaliphatic) (Table 1, entries 1–3) and the effect of the cation’s structure (Table 1, entries 3–5) on PILs’ CO_2_ sorption. At the same time, the anion nature was fixed to the tetrafluoroborate counter ion as it imparted the highest CO_2_ sorption capacity for ionic polyurethanes in our previous study [16].

Depending on the chemical nature of diisocyanate, the CO_2_ sorption values determined at 0 °C for synthesized PILs varied from 14.4 to 24.8 mg/g (Table 1, entries 1–3). The influence of the diisocyanate chemical structure on CO_2_ adsorption of ionic PURs can be ranked as follows:

**PUR2.BF_4_** (dicycloaliphatic, 24.8 mg/g) > **PUR3.BF_4_** (cycloaliphatic, 19.8 mg/g) > **PUR1.BF_4_** (aromatic, 18.3 mg/g)

This order fully correlates with the previously reported influence of diisocyanate structure on the CO_2_ sorption of ionic PUs [16]. As in the case of ionic PUs, the utilization of cycloaliphatic isophorone diisocyanate **3** in **PUR3.BF_4_** and dicycloaliphatic diisocyanate **2** in **PUR2.BF_4_** provides a higher amount of the absorbed CO_2_ in comparison with **PUR1.BF_4_** derived from aromatic diisocyanate **1**.

In this endeavor, the effect of the cation’s nature on CO_2_ sorption of ionic PURs was investigated by varying the diamines’ structure in three different PILs and keeping diisocyanate **3** and the BF_4_ anion constant (Table 1, entries 3–5). The nature of the cation in ionic PURs was found to significantly impact the CO_2_ sorption capability, which can be summarized in the following decreasing order:

**PUR3.BF_4_** (quinuclidinium, 19.8 mg/g) ≥ **PUR6.BF_4_** (diquinuclidinium, 18.3 mg/g) > **PUR5.BF_4_** (ammonium, 10.5 mg/g)

The observed superiority of the quinuclidinium-based **PUR3.BF_4_** and **PUR6.BF_4_** over the ammonium **PUR5.BF_4_** is in full agreement with the results obtained previously for the CO_2_ sorption of ionic PUs [16]. The cyclic quinuclidinium cation is much bulkier in comparison with ammonium that, in its turn, can prevent the compact packing of the polymer chains and favor the increase in free volume, thus leading to the increase in CO_2_ capture capabilities.

We have recently stated that, in the case of ionic Pus, the CO_2_ sorption does not occur by a simple physisorption mechanism, which is directly dependent on the porosity of the samples [16]. In contrast, the mechanism of CO_2_ sorption in ionic PUs was found to be complex and consists not only of the physisorption process but may also involve the specific interactions between CO_2_, ionic species and –NH–CO–O– functional groups of PILs [16,53,54]. To fulfill the aim of the study, namely to understand the influence of the additional secondary amine group on CO_2_ sorption of PILs, the ionic polyurethane **PU1.BF_4_** with a similar structure to **PUR6.BF_4_** has been additionally prepared (Table 1, entry 6). It can be seen that the transfer of –NH–CO–O– groups in **PU1.BF_4_** to –NH–CO–NH– fragments in **PUR6.BF_4_** leads to an increase in the adsorbed amount of CO_2_ (Table 1, lines 5 and 6). This can be explained by the difference in hydrogen bonding in polyureas and polyurethanes. While, in polyurethanes, the single NH group forms hydrogen bonding mainly with the C=O group of the other polymer chain, in polyureas, the second NH group remains free for the generation of additional H bonding with the CO_2_ molecule. This, in its turn, increases the capacity for CO_2_ capture in ionic PURs in comparison with ionic PUs.

Table 2 presents a comparison for CO_2_ sorption values for the best PURs and PUs selected from this study and PILs described in the literature. To the knowledge of the authors, to date, the highest CO_2_ sorption among linear PILs was demonstrated by the following polyelectrolytes: (1) positively charged cellulose (38.0 mg/g at 0.1 MPa (1 bar), 25 °C [15]); (2) cationic polyurethanes (24.8 mg/g at 0.1 MPa, 0 °C [16]); (3) poly(p-vinylbenzyl)trimethylammonium tetrafluoroborate (17.1 mg/g at 0.078 MPa, 22 °C [37]) and (4) anionic polyurethanes (16.7 mg/g at 0.1 MPa, 30 °C [40]). The results obtained for linear **PUR2.BF_4_** (24.8 mg/g at 0.1 MPa, 0 °C) can be listed among top five highest CO_2_ sorption capacities reported for PILs to date, making it a potential candidate for CO_2_ capture processes.

#### 3.3.4. Recycling Experiments

**Figure 3:**
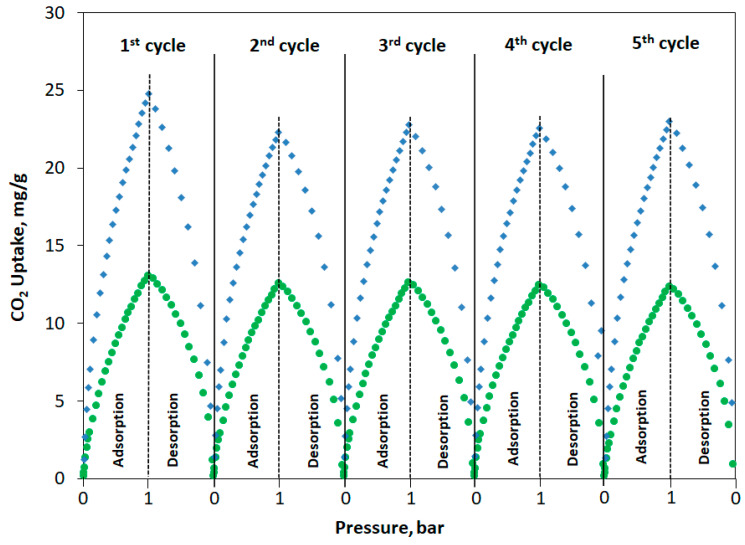
Five correlative CO_2_ adsorption/desorption isotherms (from 0.006 to 1 bar) at 0 °C for **PUR2.BF_4_** (blue squares) and **PU1.BF_4_** (green circles) carried out without degasification step between cycles.

Both ionic polyurea **PUR2.BF_4_** and ionic polyurethane **PU1.BF_4_** showed very good cyclability, with practically total reversibility in CO_2_ adsorption at 0 °C (Figure 3). After five adsorption–desorption cycles without degassing between each isotherm, the decrease in the adsorption capacity reached only 7.3% for **PUR2.BF_4_** and 5.5% for **PU1.BF_4_**. In the case of polyurea **PUR2.BF_4_**, more pronounced loss of CO_2_ adsorption capacity was observed between the first and the second cycles due to the fact that part of the CO_2_ is not desorbed in the short time (about 15 min) that the device evacuates the sample cell between consecutive measurements. This can be caused by the presence of -NH-CO-NH- functional groups with high affinity for CO_2_ (i.e., high adsorption energy) that prevent its desorption in the mild vacuum conditions between isotherms. However, between the second and fifth cycles, no decrease in the amount of adsorbed CO_2_ was observed, which could indicate that all these functional groups have been saturated. In any case, the initial adsorption capacity was recovered, for both **PUR2.BF_4_** and **PU1.BF_4_**, simply by degassing the samples at 80 °C for 4 h (results are not shown in Figure 3).

This good cyclability was also observed at atmospheric pressure and room temperature under alternating streams of N_2_ and CO_2_ in a thermobalance (Figure S3). Under the conditions indicated in the Materials and Methods, both samples **PUR2.BF_4_** and **PU1.BF_4_** reached saturation in less than 1 h and adsorbed 13.8 and 7.7 mg/g of CO_2_, respectively. Desorption under N_2_ flow was slower than adsorption and, after 2 or 3 h, complete desorption was not achieved. However, saturation was reached at the same value of 7.7 mg/g for **PU1.BF_4_** and at a slightly higher value (14.2 mg/g) for **PUR2.BF_4_** when the CO_2_ stream was again passed. Total desorption of CO_2_ can be achieved by increasing the purge time up to 8 h for **PU1.BF_4_**, but, in the case of **PUR2.BF_4_**, longer times are needed because incomplete desorption occurred in this time (see Figure S3). This is in agreement with what was discussed above (Figure 3) on the loss of adsorption capacity between the first and second adsorption–desorption cycles due to the presence of some functional groups (i.e., -NH-CO-NH- groups) with high affinity for CO_2_. Therefore, in the case of **PUR2.BF_4_**, stronger conditions are needed to desorb all the CO_2_ (e.g., longer time or higher temperature). It should be noted that both samples showed very good CO_2_/N_2_ selectivity, as demonstrated by the good cyclability in the thermobalance experiment.

### 3.4. Future Outlook

For the combustion of fossil fuels, the content of CO_2_ in flue gas varies from 10 to 15%. The other components are N_2_ (73–77%), O_2_ (4–5%), H_2_O (6–9%), CO (ppm quantity) and NOx (ppm quantity). In the case of natural gas, the main components are CH_4_, CO_2_, N_2_ and a small amount of hydrocarbons [42]. Hence, the application of PILs as well as any other material as CO_2_ sorbents will face mainly the problem of CO_2_ separation from N_2_ and from CH_4_ because these are the gases that are in greater proportion in these gaseous streams. Another problem that can arise from a practical point of view is the presence of water vapor. This is especially important in the case of adsorbents having a significant number of polar groups that will have a high affinity for water.

As demonstrated in Figure S3, PILs synthesized in the present work show very good CO_2_/N_2_ selectivity in the thermobalance experiment. It was noticed that **PUR1.BF_4_**–**PUR6.BF_4_** can also absorb some H_2_O upon long storage at room temperature. However, as in the case of ILs bearing NH_2_ groups in the side chain [57,58], the presence of traces of water in PURs will only increase the CO_2_ sorption capacity. Although the results presented here can serve as a proof-of-concept and are quite promising for sustainable CO_2_ capture, PILs, to be applied in an industrial process, should be further tested under more representative postcombustion conditions and natural gas purification; this will be the subject of our future investigations.

## 4. Conclusions

The aim of this study was to synthesize a new class of PILs, namely ionic polyureas, and to evaluate their potential for CO_2_ capture. To expand the chemistry of PILs, a synthetic route for the preparation of two ionic diamines bearing quinuclidinium cations and distinguished by simplicity and high yields was suggested. Their condensation with commercial diisocyanates and subsequent ion exchange reaction with KBF_4_ afforded series of high molecular weight (M_w_ = 12.0–173.5 × 10^3^ g/mol) and thermally stable (T_onset_ = 195–265 °C) ionic polyureas.

All synthesized PILs represent novel materials and differ by the structure of diisocyanate, the nature and the quantity of cations. All these factors were found to affect the heat resistance and the CO_2_ sorption properties of the polymers. **PUR1.BF_4_** based solely on aromatic monomers demonstrated the highest glass transition temperature (T_g_ = 286 °C), while aliphatic **PUR5.BF_4_** and **PUR6.BF_4_** showed the lowest ones (T_g_ = 153 and 209 °C, respectively). From comparative evaluation, it becomes evident that aliphatic 4,4′-methylene-bis(cyclohexyl isocyanate) imparts the highest CO_2_ capture capacity to respective ionic polyureas. The observed superiority of the quinuclidinium-based **PUR3.BF4** and **PUR6.BF4** over the ammonium **PUR5.BF4** was in full agreement with the results obtained previously for the CO_2_ sorption of ionic polyurethanes. The addition of the second quinuclidinium cation did not significantly affect the CO_2_ sorption of PILs. The comparison of structurally similar ionic polyurea **PUR6.BF_4_** and polyurethane **PU1.BF_4_** revealed that the transfer from –NH–CO–O– groups to –NH–CO–NH– fragments leads to an increase in the adsorbed amount of CO_2_, which was explained by the presence of the additional amine group and the possibility of hydrogen bonding CO_2_ molecules. Finally, these materials presented very good cyclability both in consecutive cycles of adsorption and desorption between vacuum and atmospheric pressure at 0ºC and in alternating streams of N_2_ and CO_2_ at room temperature. This last result also proves good CO_2_/N_2_ selectivity.

To conclude, the demonstrated results present a new sustainable CO_2_ capture option and add important value to novel design strategies for improving the sorption properties of PILs and the creation of novel effective CO_2_ sorbents.

## Figures and Tables

**Table 1:** Selected properties of ionic PURs and PUs.

No	Abbrev.	Poly(Ionic Liquid)	M_w_, (g/mol) ^a^	M_w_/M_n_^a^	T_g_ (°C) ^b^	T_onset_ (°C) ^c^	T_onset_ (°C) ^d^	CO_2_ Uptake, (mg/g) ^e^
		ionic polyureas (PURs)						
1	**PUR1.BF_4_**	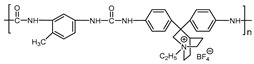	12,000	1.42	286	240	250	18.3
2	**PUR2.BF_4_**	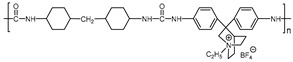	15,800	1.61	271	230	240	24.8
3	**PUR3.BF_4_**	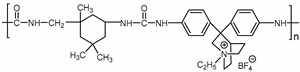	101,500	4.12	276	240	255	19.8
4	**PUR5.BF_4_**	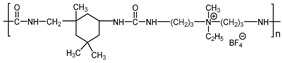	173,500	2.32	153	195	240	10.5
5	**PUR6.BF_4_**	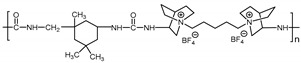	39,000	1.97	209	260	265	18.1
		ionic polyurethane (PU)						
6 ^f^	**PU1.BF_4_**	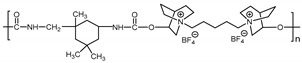	8500	1.51	195	265	265	13.1

^a^ By GPC in 0.1 M solution of NH_4_BF_4_ in DMF; ^b^ By TMA; ^c^ By TGA in air; ^d^ By TGA in N_2_ atmosphere; ^e^ 0 °C, 1 bar; ^f^ For comparison.

**Table 2:** Comparison of the CO_2_ absorption data for the best PILs reported in the literature and for those obtained in the present work.

N	PIL	Structure	CO_2_ Sorption (mg/g)	Conditions (P, T)	Reference
1	**CL-TBA**	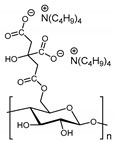	44.0	0.1 MPa, 25 °C	[55]
2	**[CelEt3N][PF_6_]**	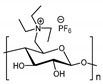	38.0	0.1 MPa, 25 °C	[15]
3	**PIL-8.1.BF_4_**	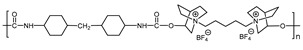	24.8	0.1 MPa, 0 °C	[16]
4	**PUR2.** **BF_4_**	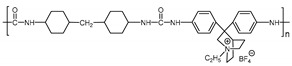	24.8	0.1 MPa, 0 °C	This work
5	**Mesoporous PIL**	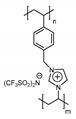	20.2	0.1 MPa, 0 °C	[20]
6	**PUR3.** **BF_4_**	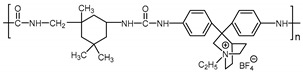	19.8	0.1 MPa, 0 °C	This work
7	**PIL-8.1.CH_3_COO**	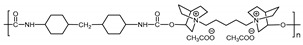	18.3	0.1 MPa, 0 °C	[16]
8	**PUR1.** **BF_4_**	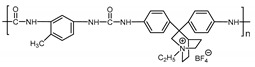	18.3	0.1 MPa, 0 °C	This work
9	**PUR6.** **BF_4_**	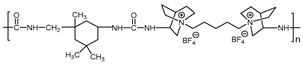	18.1	0.1 MPa, 0 °C	This work
10	**Meso-SiO_2_- P[VBTMA][BF_4_]**	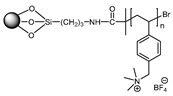	17.7	0.1 MPa, 30 °C	[25]
11	**P[VBTMA][BF_4_]**	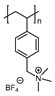	17.1	0.0078 MPa, 22 °C	[37]
12	**Meso-SiO_2_- P[VBTMA][ PF_6_]**	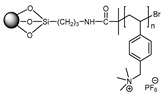	16.7	0.1 MPa, 30 °C	[25]
13	**PU-TABPU-TAB**	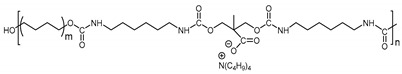	16.1	0.0082 MPa, 30 °C	[40]
14	**P[VBTMA][ PF_6_]**	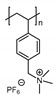	14.6	0.0078 MPa, 22 °C	[37]
15	**P[MATMA][BF_4_]**	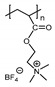	14.4	0.0078 MPa, 22 °C	[37]
16	**P[(AMIM) BF_4_-AN]**	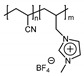	14.3	0.1 MPa, 0 °C	[56]
17	**PU1.** **BF_4_**	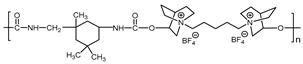	13.1	0.1 MPa, 0 °C	This work
18	**PIL-8.1.NO_3_**	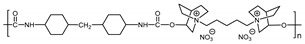	13.1	0.1 MPa, 0 °C	[16]
19	**P[BIEMA]** **[CH_3_COO]**	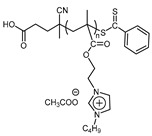	12.5	0.1 MPa, 25 °C	[19]
20	**P[VBTEA][ PF_6_]**	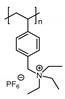	10.4	0.1 MPa, 25 °C	[21]
21	**P[VBTEA][BF_4_]**	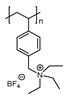	6.9	0.0078 MPa, 22 °C	[37]
22	**P[VBMI][BF_4_]**	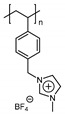	4.6	0.0078 MPa, 22 °C	[14]

## Supplementary Materials

The following are available online at https://www.mdpi.com/2077-0375/10/9/240/s1, Explanations of the choice of non-ideality factor for CO_2_ sorption calculations; Figure S1: (a) ^1^H, (b) HMBC and (c) ^19^F NMR spectra of polyurea **PUR6.BF_4_** (25 °C, DMSO-*d_6_*); Figure S2: FT-IR spectrum of polyurea **PUR6.BF_4_**; Figure S3: CO_2_ sorption/desorption tests for **PUR2.BF_4_** and **PU1.****BF_4_** at atmospheric pressure and room temperature under alternating streams of N_2_ and CO_2_ in a thermobalance.
