# Supplementary material for: Ionic Polyureas—A Novel Subclass of Poly(Ionic Liquid)s for CO2 Capture
Source: Membranes (Basel). 2020 Sep 18;10(9):240. doi: 10.3390/membranes10090240 (PMC7558175; doi:10.3390/membranes10090240)
Supplement: Supplementary file 1 [file membranes-10-00240-s001.pdf]

membranes

Supplementary Materials: Ionic polyureas - a novel subclass of poly(ionic liquid)s for CO_2 capture

Supplementary Materials

1.1. Explanations on the choice of non-ideality factor for CO_2 sorption calculations

Most gases can be considered as ideal ones at sub-atmospheric pressures. In the case of CO_2 at 0 ℃, its density at 760 mmHg is 0.04462 mol/l using the ideal equation of state (EOS) and 0.04492 mol/l using the Helmholtz EOS proposed by Span and Wagner [1], which is the equation recommended by the National Institute of Standards and Technologies of USA (NIST) for CO_2 at these conditions.

Commercial sub-atmospheric devices for measuring adsorption isotherms, in general, do not use real EOS (e.g. Helmholtz equation) to calculate the gas density at each measured pressure (due the very low difference between the ideal and real density of gases at low pressures), but they use the non-ideality factor which is calculated at 760 mmHg. In the present case, we have used a non-ideality factor of -8.93 10^{-6} $mmHg^{-1}$ calculated using the Helmholtz equation [1].

The ideal gas law can be extended to real gases introducing the compressibility factor (Z):

$$PV = ZnRT \qquad \text{Equation (1)}$$

, where P, V, n, R and T are the pressure, volume, number of moles, the gas constant and temperature, respectively. Consequently, the extent of the deviation from the ideal gas law exhibited by a gas is (Z – 1), which is related to P through the non-ideality factor (α) as:

$$Z - 1 = \alpha P \quad \text{or} \quad Z = 1 + \alpha P \qquad \text{Equation (2)}$$

Note that α in Equation 2 is independent of pressure. Therefore, this non-ideality factor (**-8.93×10^{-6} $mmHg^{-1}$**) is used at each tested pressure for calculating the number of moles through the Equation 3.

$$PV = (1 + \alpha P)nRT \qquad \text{Equation (3)}$$

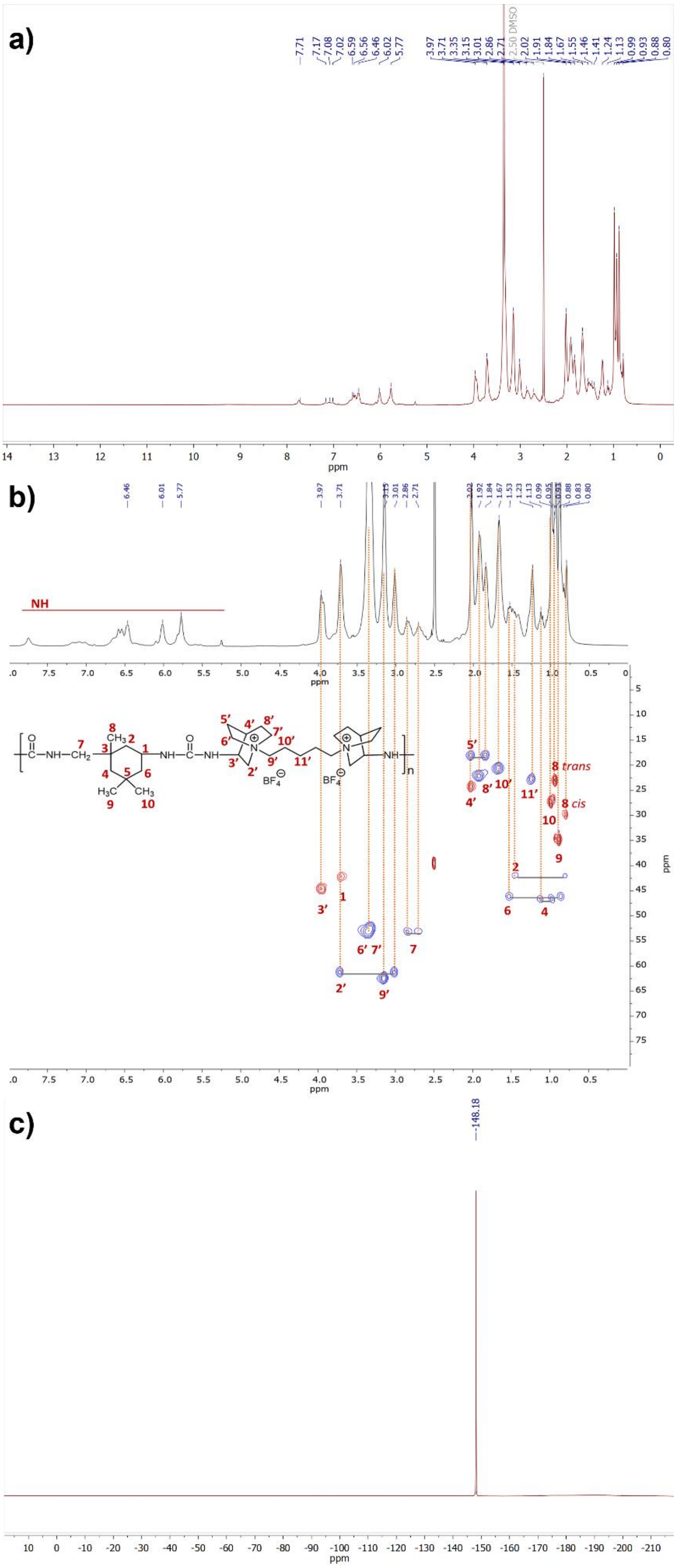

Figure S1. (**a**) ^{1}H, (**b**) HMBC and (**c**) ^{19}F NMR spectra of polyurea **PUR6.BF$_4$** (25°C, DMSO-*d*$_6$).

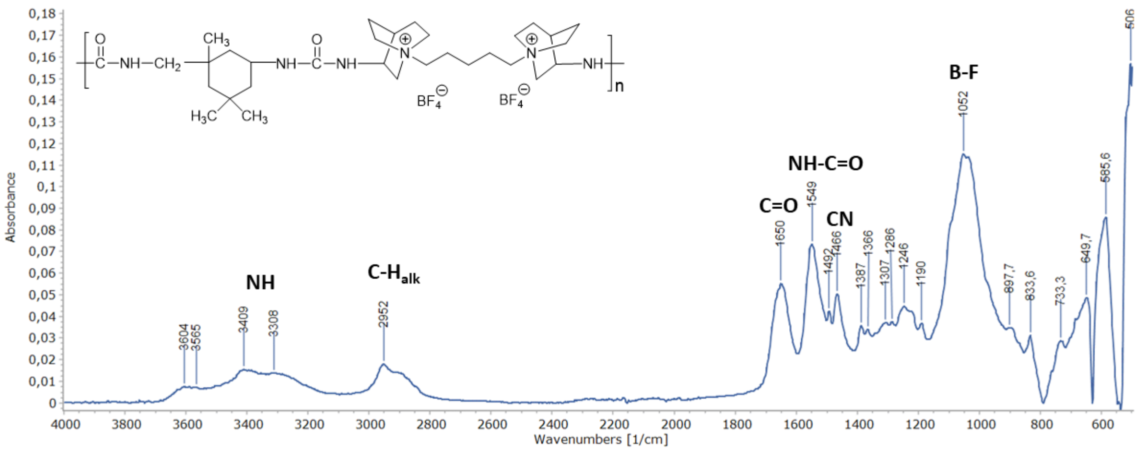

Figure S2. FT-IR spectrum of polyurea **PUR6.BF**$_4$.

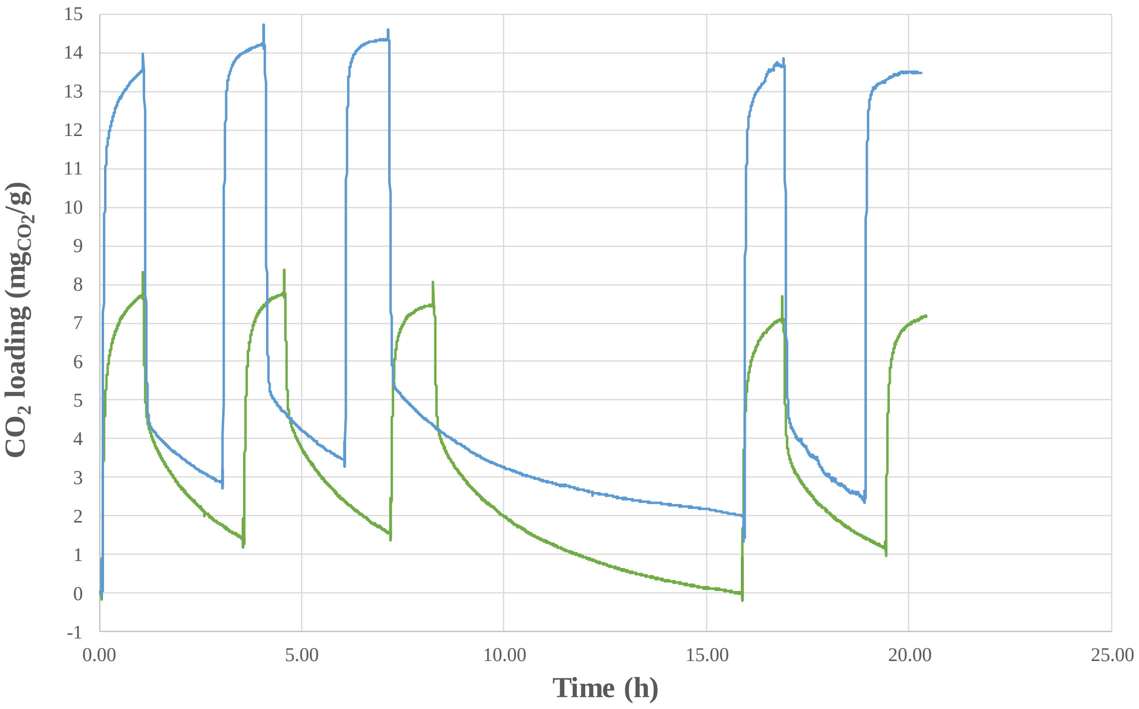

Figure S3. CO_2 sorption/desorption tests for **PU1.BF**$_4$ (blue) and **PU1.BF**$_4$ (green) at atmospheric pressure and room temperature under alternating streams of N_2 and CO_2 in a thermobalance.

References

1. Span, R.; Wagner, W. A New Equation of State for Carbon Dioxide Covering the Fluid Region from the Triple-Point Temperature to 1100 K at Pressures up to 800 MPa. *J. Phys. Chem. Ref. Data* **1996**, *25*, 1509–1596, doi:10.1063/1.555991.

© 2020 by the authors. Submitted for possible open access publication under the terms and conditions of the Creative Commons Attribution (CC BY) license (http://creativecommons.org/licenses/by/4.0/).
